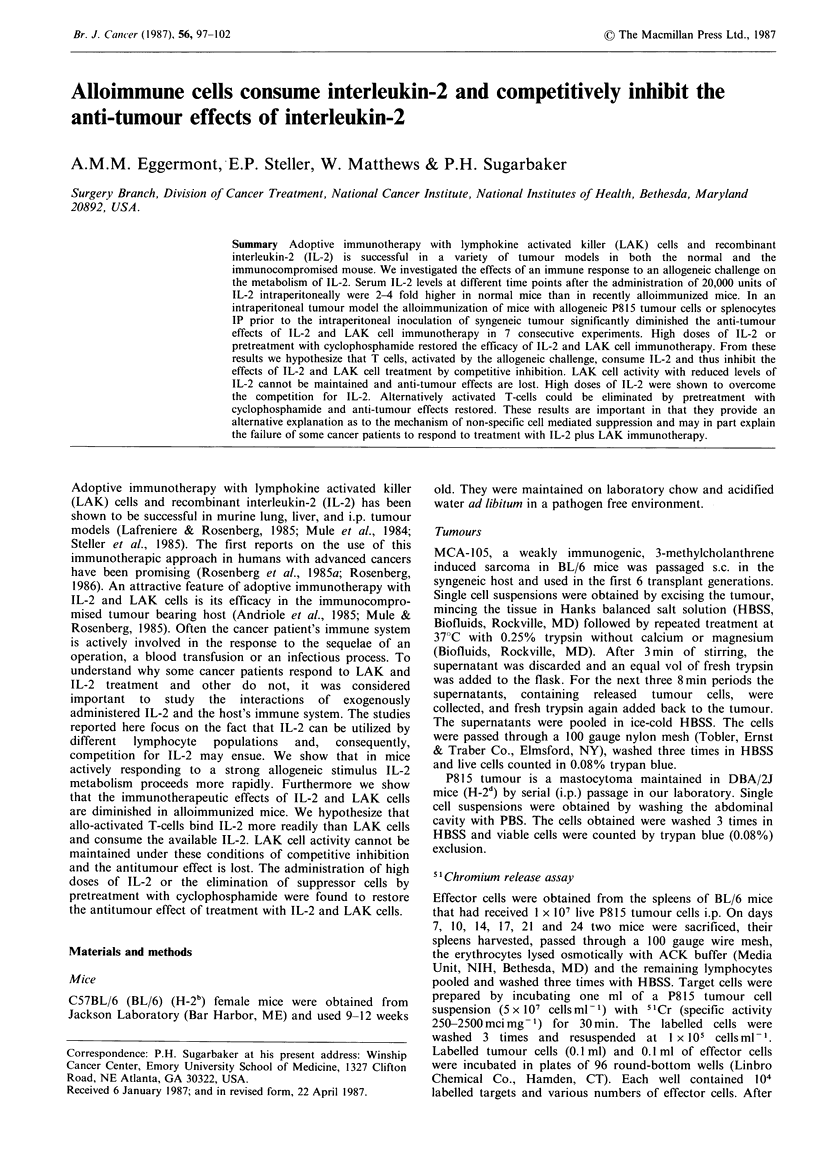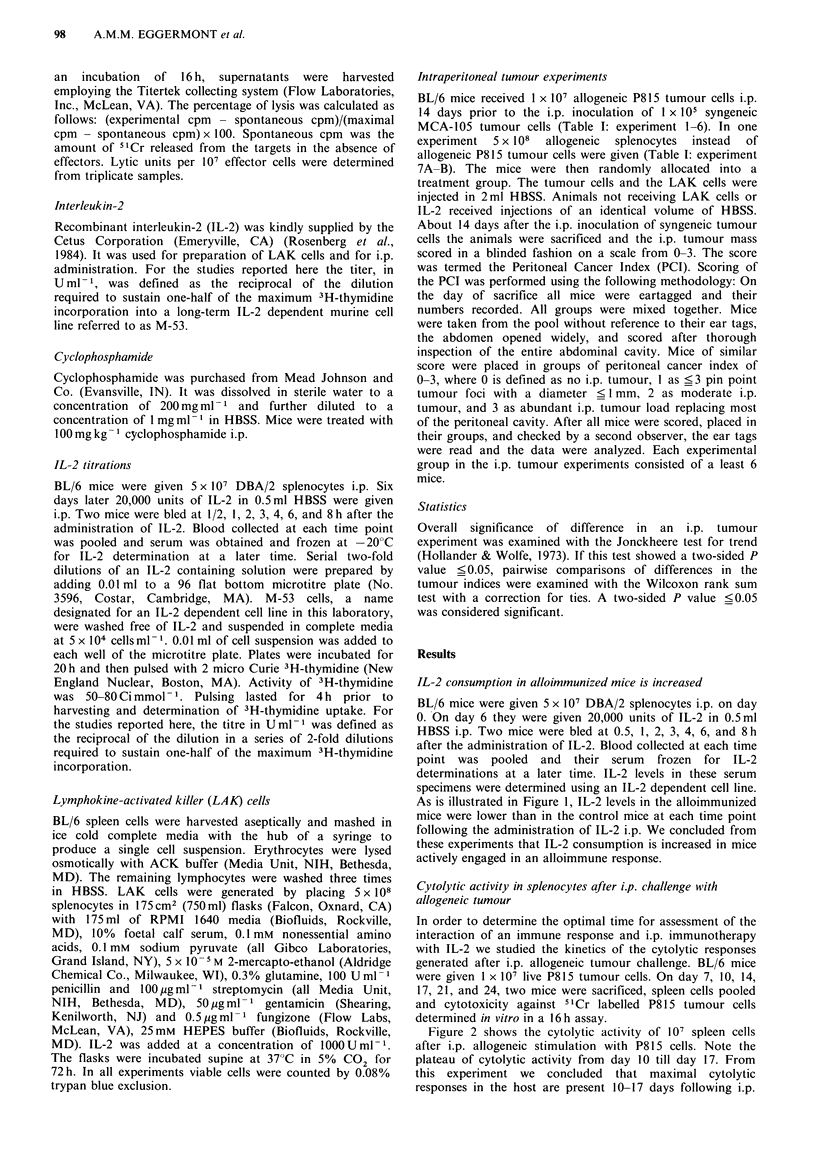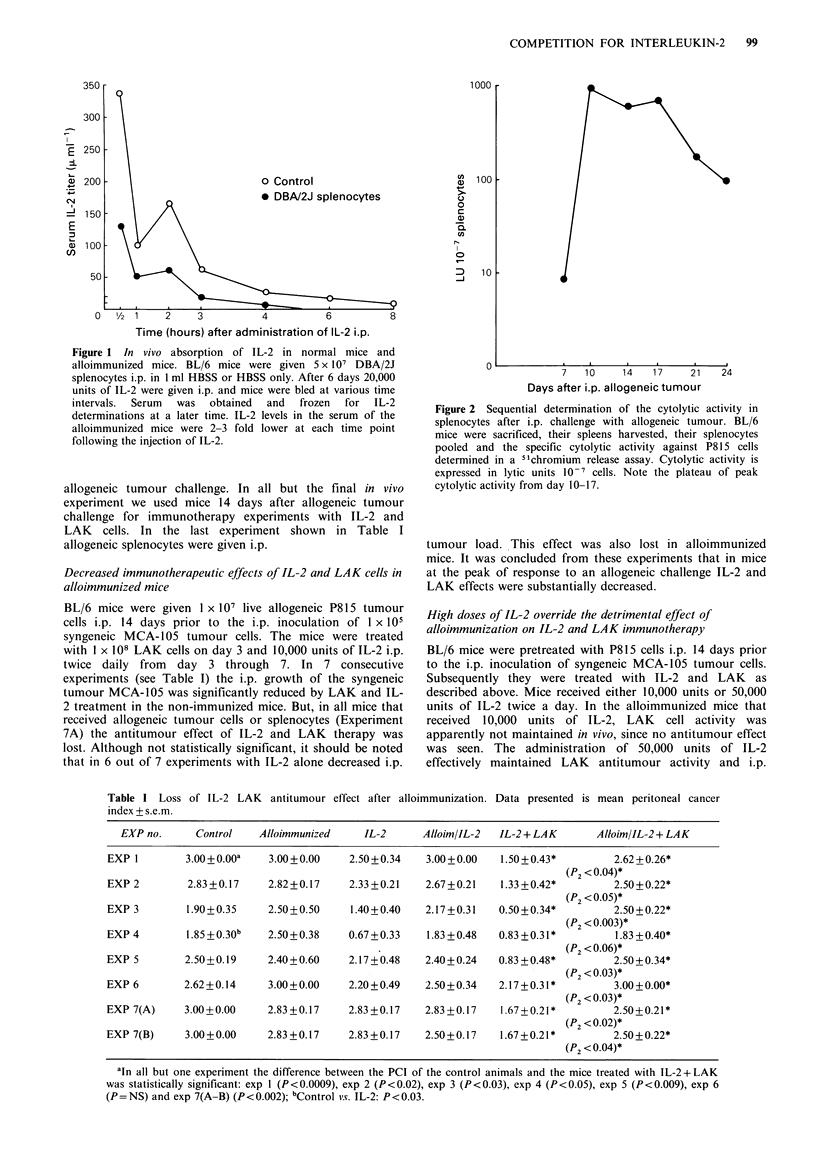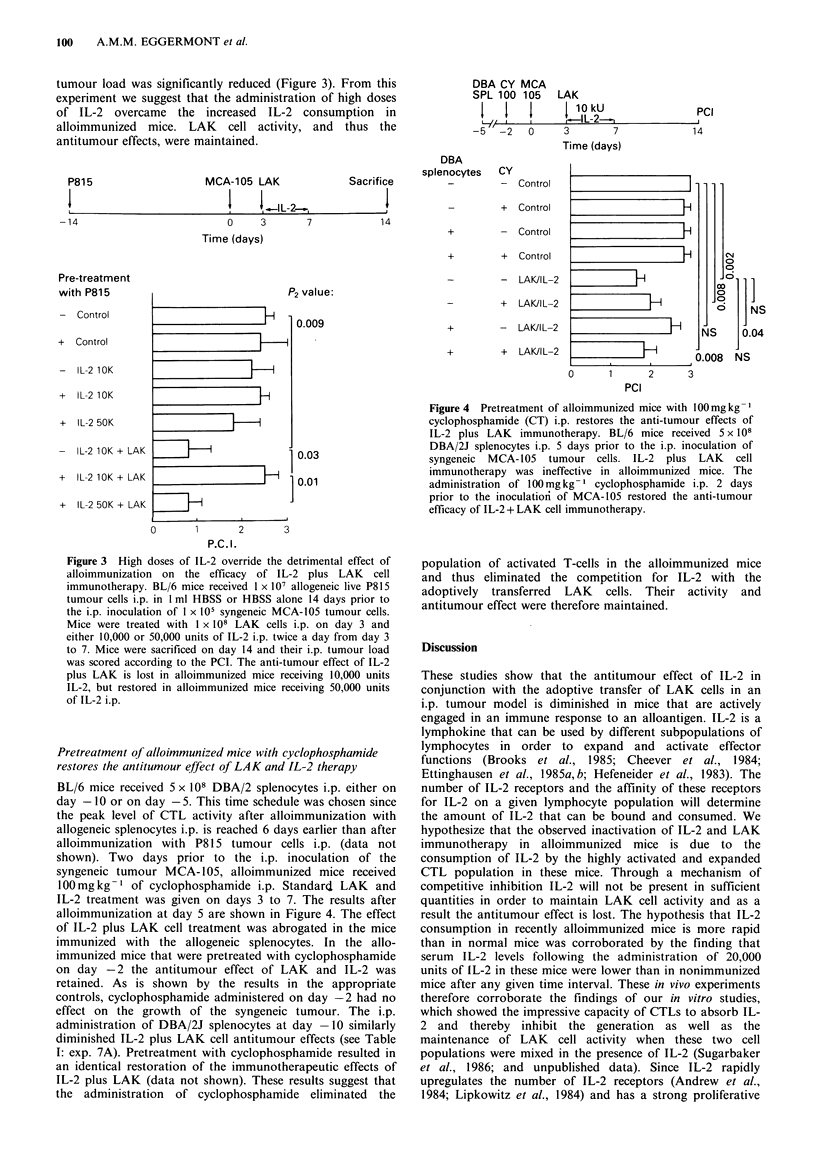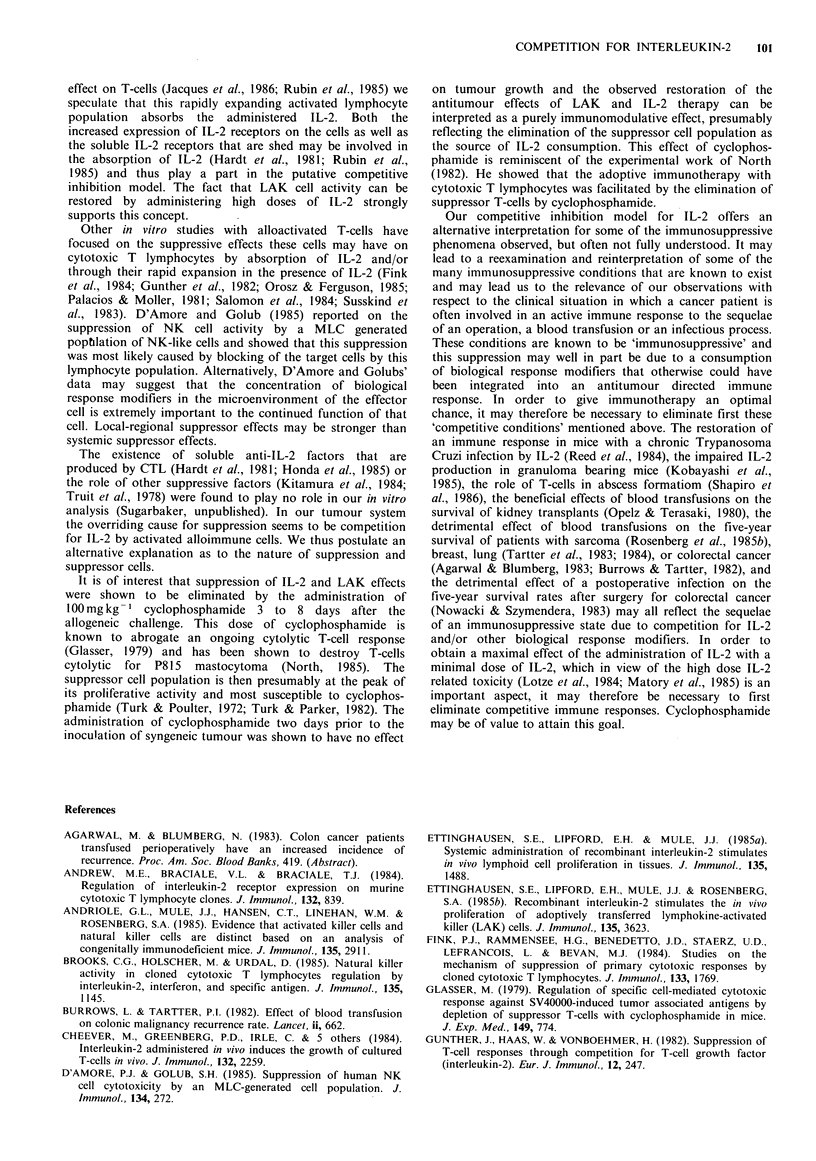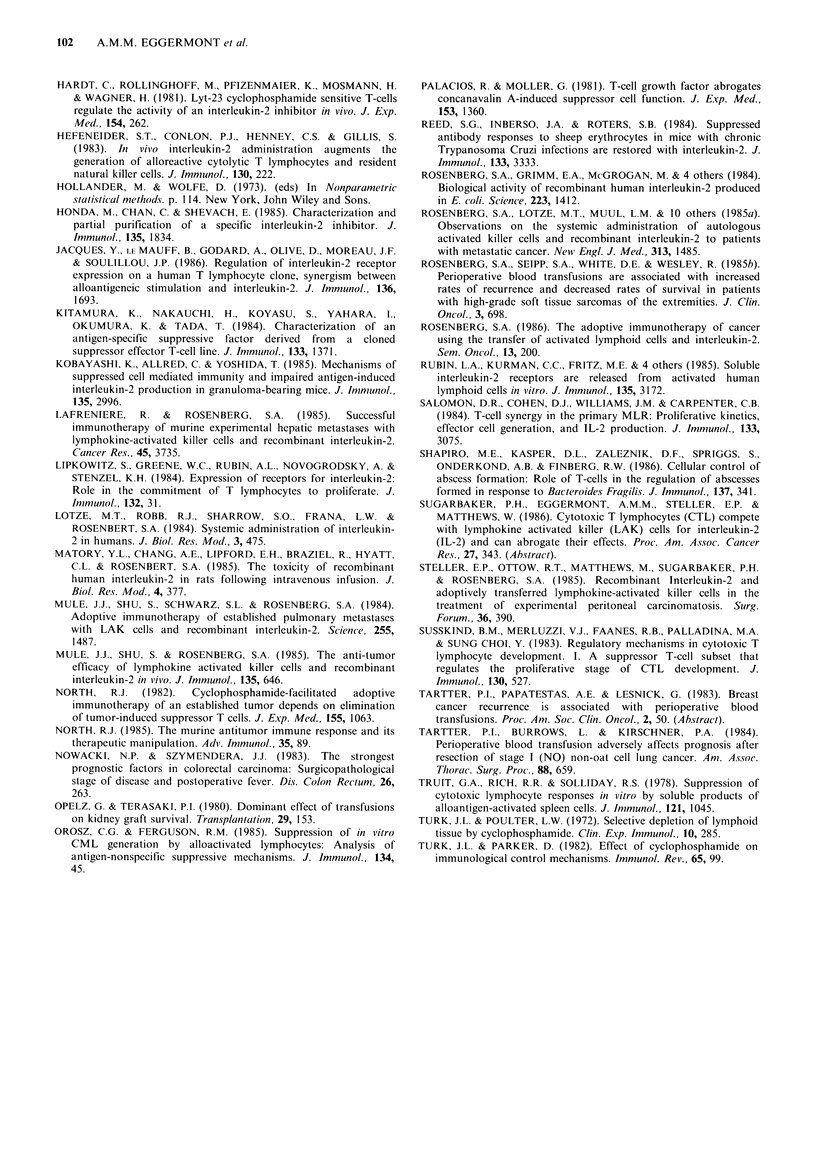# Alloimmune cells consume interleukin-2 and competitively inhibit the anti-tumour effects of interleukin-2.

**DOI:** 10.1038/bjc.1987.164

**Published:** 1987-08

**Authors:** A. M. Eggermont, E. P. Steller, W. Matthews, P. H. Sugarbaker

**Affiliations:** Surgery Branch, National Cancer Institute, Bethesda, Maryland 20892.

## Abstract

Adoptive immunotherapy with lymphokine activated killer (LAK) cells and recombinant interleukin-2 (IL-2) is successful in a variety of tumour models in both the normal and the immunocompromised mouse. We investigated the effects of an immune response to an allogeneic challenge on the metabolism of IL-2. Serum IL-2 levels at different time points after the administration of 20,000 units of IL-2 intraperitoneally were 2-4 fold higher in normal mice than in recently alloimmunized mice. In an intraperitoneal tumour model the alloimmunization of mice with allogeneic P815 tumour cells or splenocytes IP prior to the intraperitoneal inoculation of syngeneic tumour significantly diminished the anti-tumour effects of IL-2 and LAK cell immunotherapy in 7 consecutive experiments. High doses of IL-2 or pretreatment with cyclophosphamide restored the efficacy of IL-2 and LAK cell immunotherapy. From these results we hypothesize that T cells, activated by the allogeneic challenge, consume IL-2 and thus inhibit the effects of IL-2 and LAK cell treatment by competitive inhibition. LAK cell activity with reduced levels of IL-2 cannot be maintained and anti-tumour effects are lost. High doses of IL-2 were shown to overcome the competition for IL-2. Alternatively activated T-cells could be eliminated by pretreatment with cyclophosphamide and anti-tumour effects restored. These results are important in that they provide an alternative explanation as to the mechanism of non-specific cell mediated suppression and may in part explain the failure of some cancer patients to respond to treatment with IL-2 plus LAK immunotherapy.


Br. J. Cancer (1987), 56, 97–102 © The Macmillan Press Ltd., 1987

### Alloimmune cells consume interleukin-2 and competitively inhibit the anti-tumour effects of interleukin-2

A.M.M. Eggermont, E.P. Steller, W. Matthews & P.H. Sugarbaker

*Surgery Branch, Division of Cancer Treatment, National Cancer Institute, National Institutes of Health, Bethesda, Maryland 20892, USA.*

**Summary** Adoptive immunotherapy with lymphokine activated killer (LAK) cells and recombinant interleukin-2 (IL-2) is successful in a variety of tumour models in both the normal and the immunocompromised mouse. We investigated the effects of an immune response to an allogeneic challenge on the metabolism of IL-2. Serum IL-2 levels at different time points after the administration of 20,000 units of IL-2 intraperitoneally were 2–4 fold higher in normal mice than in recently alloimmunized mice. In an intraperitoneal tumour model the alloimmunization of mice with allogeneic P815 tumour cells or splenocytes IP prior to the intraperitoneal inoculation of syngeneic tumour significantly diminished the anti-tumour effects of IL-2 and LAK cell immunotherapy in 7 consecutive experiments. High doses of IL-2 or pretreatment with cyclophosphamide restored the efficacy of IL-2 and LAK cell immunotherapy. From these results we hypothesize that T cells, activated by the allogeneic challenge, consume IL-2 and thus inhibit the effects of IL-2 and LAK cell treatment by competitive inhibition. LAK cell activity with reduced levels of IL-2 cannot be maintained and anti-tumour effects are lost. High doses of IL-2 were shown to overcome the competition for IL-2. Alternatively activated T-cells could be eliminated by pretreatment with cyclophosphamide and anti-tumour effects restored. These results are important in that they provide an alternative explanation as to the mechanism of non-specific cell mediated suppression and may in part explain the failure of some cancer patients to respond to treatment with IL-2 plus LAK immunotherapy.

Adoptive immunotherapy with lymphokine activated killer (LAK) cells and recombinant interleukin-2 (IL-2) has been shown to be successful in murine lung, liver, and i.p. tumour models (Lafreniere & Rosenberg, 1985; Mule *et al.*, 1984; Steller *et al.*, 1985). The first reports on the use of this immunotherapic approach in humans with advanced cancers have been promising (Rosenberg *et al.*, 1985*a*; Rosenberg, 1986). An attractive feature of adoptive immunotherapy with IL-2 and LAK cells is its efficacy in the immunocompromised tumour bearing host (Andriole *et al.*, 1985; Mule & Rosenberg, 1985). Often the cancer patient's immune system is actively involved in the response to the sequelae of an operation, a blood transfusion or an infectious process. To understand why some cancer patients respond to LAK and IL-2 treatment and other do not, it was considered important to study the interactions of exogenously administered IL-2 and the host's immune system. The studies reported here focus on the fact that IL-2 can be utilized by different lymphocyte populations and, consequently, competition for IL-2 may ensue. We show that in mice actively responding to a strong allogeneic stimulus IL-2 metabolism proceeds more rapidly. Furthermore we show that the immunotherapeutic effects of IL-2 and LAK cells are diminished in alloimmunized mice. We hypothesize that allo-activated T-cells bind IL-2 more readily than LAK cells and consume the available IL-2. LAK cell activity cannot be maintained under these conditions of competitive inhibition and the antitumour effect is lost. The administration of high doses of IL-2 or the elimination of suppressor cells by pretreatment with cyclophosphamide were found to restore the antitumour effect of treatment with IL-2 and LAK cells.

### Materials and methods

### *Mice*

C57BL/6 (BL/6) (H-$2^b$) female mice were obtained from Jackson Laboratory (Bar Harbor, ME) and used 9–12 weeks old. They were maintained on laboratory chow and acidified water *ad libitum* in a pathogen free environment.

### *Tumours*

MCA-105, a weakly immunogenic, 3-methylcholanthrene induced sarcoma in BL/6 mice was passaged s.c. in the syngeneic host and used in the first 6 transplant generations. Single cell suspensions were obtained by excising the tumour, mincing the tissue in Hanks balanced salt solution (HBSS, Biofluids, Rockville, MD) followed by repeated treatment at 37°C with 0.25% trypsin without calcium or magnesium (Biofluids, Rockville, MD). After 3 min of stirring, the supernatant was discarded and an equal vol of fresh trypsin was added to the flask. For the next three 8 min periods the supernatants, containing released tumour cells, were collected, and fresh trypsin again added back to the tumour. The supernatants were pooled in ice-cold HBSS. The cells were passed through a 100 gauge nylon mesh (Tobler, Ernst & Traber Co., Elmsford, NY), washed three times in HBSS and live cells counted in 0.08% trypan blue.

P815 tumour is a mastocytoma maintained in DBA/2J mice (H-$2^d$) by serial (i.p.) passage in our laboratory. Single cell suspensions were obtained by washing the abdominal cavity with PBS. The cells obtained were washed 3 times in HBSS and viable cells were counted by trypan blue (0.08%) exclusion.

### *$^{51}$Chromium release assay*

Effector cells were obtained from the spleens of BL/6 mice that had received $1\times10^7$ live P815 tumour cells i.p. On days 7, 10, 14, 17, 21 and 24 two mice were sacrificed, their spleens harvested, passed through a 100 gauge wire mesh, the erythrocytes lysed osmotically with ACK buffer (Media Unit, NIH, Bethesda, MD) and the remaining lymphocytes pooled and washed three times with HBSS. Target cells were prepared by incubating one ml of a P815 tumour cell suspension ($5\times10^7$ cells ml$^{-1}$) with $^{51}$Cr (specific activity 250–2500 mci mg$^{-1}$) for 30 min. The labelled cells were washed 3 times and resuspended at $1\times10^5$ cells ml$^{-1}$. Labelled tumour cells (0.1 ml) and 0.1 ml of effector cells were incubated in plates of 96 round-bottom wells (Linbro Chemical Co., Hamden, CT). Each well contained $10^4$ labelled targets and various numbers of effector cells. After

Correspondence: P.H. Sugarbaker at his present address: Winship Cancer Center, Emory University School of Medicine, 1327 Clifton Road, NE Atlanta, GA 30322, USA.
Received 6 January 1987; and in revised form, 22 April 1987.

an incubation of 16 h, supernatants were harvested employing the Titertek collecting system (Flow Laboratories, Inc., McLean, VA). The percentage of lysis was calculated as follows: (experimental cpm − spontaneous cpm)/(maximal cpm − spontaneous cpm) × 100. Spontaneous cpm was the amount of $^{51}Cr$ released from the targets in the absence of effectors. Lytic units per $10^7$ effector cells were determined from triplicate samples.

*Interleukin-2*

Recombinant interleukin-2 (IL-2) was kindly supplied by the Cetus Corporation (Emeryville, CA) (Rosenberg *et al.*, 1984). It was used for preparation of LAK cells and for i.p. administration. For the studies reported here the titer, in U ml$^{-1}$, was defined as the reciprocal of the dilution required to sustain one-half of the maximum $^3$H-thymidine incorporation into a long-term IL-2 dependent murine cell line referred to as M-53.

*Cyclophosphamide*

Cyclophosphamide was purchased from Mead Johnson and Co. (Evansville, IN). It was dissolved in sterile water to a concentration of 200 mg ml$^{-1}$ and further diluted to a concentration of 1 mg ml$^{-1}$ in HBSS. Mice were treated with 100 mg kg$^{-1}$ cyclophosphamide i.p.

*IL-2 titrations*

BL/6 mice were given $5 \times 10^7$ DBA/2 splenocytes i.p. Six days later 20,000 units of IL-2 in 0.5 ml HBSS were given i.p. Two mice were bled at 1/2, 1, 2, 3, 4, 6, and 8 h after the administration of IL-2. Blood collected at each time point was pooled and serum was obtained and frozen at −20°C for IL-2 determination at a later time. Serial two-fold dilutions of an IL-2 containing solution were prepared by adding 0.01 ml to a 96 flat bottom microtitre plate (No. 3596, Costar, Cambridge, MA). M-53 cells, a name designated for an IL-2 dependent cell line in this laboratory, were washed free of IL-2 and suspended in complete media at $5 \times 10^4$ cells ml$^{-1}$. 0.01 ml of cell suspension was added to each well of the microtitre plate. Plates were incubated for 20 h and then pulsed with 2 micro Curie $^3$H-thymidine (New England Nuclear, Boston, MA). Activity of $^3$H-thymidine was 50–80 Ci mmol$^{-1}$. Pulsing lasted for 4 h prior to harvesting and determination of $^3$H-thymidine uptake. For the studies reported here, the titre in U ml$^{-1}$ was defined as the reciprocal of the dilution in a series of 2-fold dilutions required to sustain one-half of the maximum $^3$H-thymidine incorporation.

*Lymphokine-activated killer (LAK) cells*

BL/6 spleen cells were harvested aseptically and mashed in ice cold complete media with the hub of a syringe to produce a single cell suspension. Erythrocytes were lysed osmotically with ACK buffer (Media Unit, NIH, Bethesda, MD). The remaining lymphocytes were washed three times in HBSS. LAK cells were generated by placing $5 \times 10^8$ splenocytes in 175 cm$^2$ (750 ml) flasks (Falcon, Oxnard, CA) with 175 ml of RPMI 1640 media (Biofluids, Rockville, MD), 10% foetal calf serum, 0.1 mM nonessential amino acids, 0.1 mM sodium pyruvate (all Gibco Laboratories, Grand Island, NY), $5 \times 10^{-5}$ M 2-mercapto-ethanol (Aldridge Chemical Co., Milwaukee, WI), 0.3% glutamine, 100 U ml$^{-1}$ penicillin and 100 $\mu$g ml$^{-1}$ streptomycin (all Media Unit, NIH, Bethesda, MD), 50 $\mu$g ml$^{-1}$ gentamicin (Shearing, Kenilworth, NJ) and 0.5 $\mu$g ml$^{-1}$ fungizone (Flow Labs, McLean, VA), 25 mM HEPES buffer (Biofluids, Rockville, MD). IL-2 was added at a concentration of 1000 U ml$^{-1}$. The flasks were incubated supine at 37°C in 5% $CO_2$ for 72 h. In all experiments viable cells were counted by 0.08% trypan blue exclusion.

*Intraperitoneal tumour experiments*

BL/6 mice received $1 \times 10^7$ allogeneic P815 tumour cells i.p. 14 days prior to the i.p. inoculation of $1 \times 10^5$ syngeneic MCA-105 tumour cells (Table I: experiment 1–6). In one experiment $5 \times 10^8$ allogeneic splenocytes instead of allogeneic P815 tumour cells were given (Table I: experiment 7A–B). The mice were then randomly allocated into a treatment group. The tumour cells and the LAK cells were injected in 2 ml HBSS. Animals not receiving LAK cells or IL-2 received injections of an identical volume of HBSS. About 14 days after the i.p. inoculation of syngeneic tumour cells the animals were sacrificed and the i.p. tumour mass scored in a blinded fashion on a scale from 0–3. The score was termed the Peritoneal Cancer Index (PCI). Scoring of the PCI was performed using the following methodology: On the day of sacrifice all mice were eartagged and their numbers recorded. All groups were mixed together. Mice were taken from the pool without reference to their ear tags, the abdomen opened widely, and scored after thorough inspection of the entire abdominal cavity. Mice of similar score were placed in groups of peritoneal cancer index of 0–3, where 0 is defined as no i.p. tumour, 1 as ≦3 pin point tumour foci with a diameter ≦1 mm, 2 as moderate i.p. tumour, and 3 as abundant i.p. tumour load replacing most of the peritoneal cavity. After all mice were scored, placed in their groups, and checked by a second observer, the ear tags were read and the data were analyzed. Each experimental group in the i.p. tumour experiments consisted of a least 6 mice.

*Statistics*

Overall significance of difference in an i.p. tumour experiment was examined with the Jonckheere test for trend (Hollander & Wolfe, 1973). If this test showed a two-sided $P$ value ≦0.05, pairwise comparisons of differences in the tumour indices were examined with the Wilcoxon rank sum test with a correction for ties. A two-sided $P$ value ≦0.05 was considered significant.

### Results

*IL-2 consumption in alloimmunized mice is increased*

BL/6 mice were given $5 \times 10^7$ DBA/2 splenocytes i.p. on day 0. On day 6 they were given 20,000 units of IL-2 in 0.5 ml HBSS i.p. Two mice were bled at 0.5, 1, 2, 3, 4, 6, and 8 h after the administration of IL-2. Blood collected at each time point was pooled and their serum frozen for IL-2 determinations at a later time. IL-2 levels in these serum specimens were determined using an IL-2 dependent cell line. As is illustrated in Figure 1, IL-2 levels in the alloimmunized mice were lower than in the control mice at each time point following the administration of IL-2 i.p. We concluded from these experiments that IL-2 consumption is increased in mice actively engaged in an alloimmune response.

*Cytolytic activity in splenocytes after i.p. challenge with allogeneic tumour*

In order to determine the optimal time for assessment of the interaction of an immune response and i.p. immunotherapy with IL-2 we studied the kinetics of the cytolytic responses generated after i.p. allogeneic tumour challenge. BL/6 mice were given $1 \times 10^7$ live P815 tumour cells. On day 7, 10, 14, 17, 21, and 24, two mice were sacrificed, spleen cells pooled and cytotoxicity against $^{51}Cr$ labelled P815 tumour cells determined *in vitro* in a 16 h assay.

Figure 2 shows the cytolytic activity of $10^7$ spleen cells after i.p. allogeneic stimulation with P815 cells. Note the plateau of cytolytic activity from day 10 till day 17. From this experiment we concluded that maximal cytolytic responses in the host are present 10–17 days following i.p.

**Figure 1** *In vivo* absorption of IL-2 in normal mice and alloimmunized mice. BL/6 mice were given $5 \times 10^7$ DBA/2J splenocytes i.p. in 1 ml HBSS or HBSS only. After 6 days 20,000 units of IL-2 were given i.p. and mice were bled at various time intervals. Serum was obtained and frozen for IL-2 determinations at a later time. IL-2 levels in the serum of the alloimmunized mice were 2–3 fold lower at each time point following the injection of IL-2.

**Figure 2** Sequential determination of the cytolytic activity in splenocytes after i.p. challenge with allogeneic tumour. BL/6 mice were sacrificed, their spleens harvested, their splenocytes pooled and the specific cytolytic activity against P815 cells determined in a $^{51}$chromium release assay. Cytolytic activity is expressed in lytic units $10^{-7}$ cells. Note the plateau of peak cytolytic activity from day 10–17.

allogeneic tumour challenge. In all but the final *in vivo* experiment we used mice 14 days after allogeneic tumour challenge for immunotherapy experiments with IL-2 and LAK cells. In the last experiment shown in Table I allogeneic splenocytes were given i.p.

### *Decreased immunotherapeutic effects of IL-2 and LAK cells in alloimmunized mice*

BL/6 mice were given $1 \times 10^7$ live allogeneic P815 tumour cells i.p. 14 days prior to the i.p. inoculation of $1 \times 10^5$ syngeneic MCA-105 tumour cells. The mice were treated with $1 \times 10^8$ LAK cells on day 3 and 10,000 units of IL-2 i.p. twice daily from day 3 through 7. In 7 consecutive experiments (see Table I) the i.p. growth of the syngeneic tumour MCA-105 was significantly reduced by LAK and IL-2 treatment in the non-immunized mice. But, in all mice that received allogeneic tumour cells or splenocytes (Experiment 7A) the antitumour effect of IL-2 and LAK therapy was lost. Although not statistically significant, it should be noted that in 6 out of 7 experiments with IL-2 alone decreased i.p. tumour load. This effect was also lost in alloimmunized mice. It was concluded from these experiments that in mice at the peak of response to an allogeneic challenge IL-2 and LAK effects were substantially decreased.

### *High doses of IL-2 override the detrimental effect of alloimmunization on IL-2 and LAK immunotherapy*

BL/6 mice were pretreated with P815 cells i.p. 14 days prior to the i.p. inoculation of syngeneic MCA-105 tumour cells. Subsequently they were treated with IL-2 and LAK as described above. Mice received either 10,000 units or 50,000 units of IL-2 twice a day. In the alloimmunized mice that received 10,000 units of IL-2, LAK cell activity was apparently not maintained *in vivo*, since no antitumour effect was seen. The administration of 50,000 units of IL-2 effectively maintained LAK antitumour activity and i.p.

**Table I** Loss of IL-2 LAK antitumour effect after alloimmunization. Data presented is mean peritoneal cancer index ± s.e.m.

| *EXP no.* | *Control* | *Alloimmunized* | *IL-2* | *Alloim/IL-2* | *IL-2 + LAK* | *Alloim/IL-2 + LAK* |
|---|---|---|---|---|---|---|
| EXP 1 | 3.00 ± 0.00[a] | 3.00 ± 0.00 | 2.50 ± 0.34 | 3.00 ± 0.00 | 1.50 ± 0.43* | 2.62 ± 0.26* ($P_2$ < 0.04)* |
| EXP 2 | 2.83 ± 0.17 | 2.82 ± 0.17 | 2.33 ± 0.21 | 2.67 ± 0.21 | 1.33 ± 0.42* | 2.50 ± 0.22* ($P_2$ < 0.05)* |
| EXP 3 | 1.90 ± 0.35 | 2.50 ± 0.50 | 1.40 ± 0.40 | 2.17 ± 0.31 | 0.50 ± 0.34* | 2.50 ± 0.22* ($P_2$ < 0.003)* |
| EXP 4 | 1.85 ± 0.30[b] | 2.50 ± 0.38 | 0.67 ± 0.33 | 1.83 ± 0.48 | 0.83 ± 0.31* | 1.83 ± 0.40* ($P_2$ < 0.06)* |
| EXP 5 | 2.50 ± 0.19 | 2.40 ± 0.60 | 2.17 ± 0.48 | 2.40 ± 0.24 | 0.83 ± 0.48* | 2.50 ± 0.34* ($P_2$ < 0.03)* |
| EXP 6 | 2.62 ± 0.14 | 3.00 ± 0.00 | 2.20 ± 0.49 | 2.50 ± 0.34 | 2.17 ± 0.31* | 3.00 ± 0.00* ($P_2$ < 0.03)* |
| EXP 7(A) | 3.00 ± 0.00 | 2.83 ± 0.17 | 2.83 ± 0.17 | 2.83 ± 0.17 | 1.67 ± 0.21* | 2.50 ± 0.21* ($P_2$ < 0.02)* |
| EXP 7(B) | 3.00 ± 0.00 | 2.83 ± 0.17 | 2.83 ± 0.17 | 2.50 ± 0.17 | 1.67 ± 0.21* | 2.50 ± 0.22* ($P_2$ < 0.04)* |

[a]In all but one experiment the difference between the PCI of the control animals and the mice treated with IL-2 + LAK was statistically significant: exp 1 ($P$ < 0.0009), exp 2 ($P$ < 0.02), exp 3 ($P$ < 0.03), exp 4 ($P$ < 0.05), exp 5 ($P$ < 0.009), exp 6 ($P$ = NS) and exp 7(A–B) ($P$ < 0.002); [b]Control *vs.* IL-2: $P$ < 0.03.

tumour load was significantly reduced (Figure 3). From this experiment we suggest that the administration of high doses of IL-2 overcame the increased IL-2 consumption in alloimmunized mice. LAK cell activity, and thus the antitumour effects, were maintained.

**Figure 3** High doses of IL-2 override the detrimental effect of alloimmunization on the efficacy of IL-2 plus LAK cell immunotherapy. BL/6 mice received $1 \times 10^7$ allogeneic live P815 tumour cells i.p. in 1 ml HBSS or HBSS alone 14 days prior to the i.p. inoculation of $1 \times 10^5$ syngeneic MCA-105 tumour cells. Mice were treated with $1 \times 10^8$ LAK cells i.p. on day 3 and either 10,000 or 50,000 units of IL-2 i.p. twice a day from day 3 to 7. Mice were sacrificed on day 14 and their i.p. tumour load was scored according to the PCI. The anti-tumour effect of IL-2 plus LAK is lost in alloimmunized mice receiving 10,000 units IL-2, but restored in alloimmunized mice receiving 50,000 units of IL-2 i.p.

### *Pretreatment of alloimmunized mice with cyclophosphamide restores the antitumour effect of LAK and IL-2 therapy*

BL/6 mice received $5 \times 10^8$ DBA/2 splenocytes i.p. either on day $-10$ or on day $-5$. This time schedule was chosen since the peak level of CTL activity after alloimmunization with allogeneic splenocytes i.p. is reached 6 days earlier than after alloimmunization with P815 tumour cells i.p. (data not shown). Two days prior to the i.p. inoculation of the syngeneic tumour MCA-105, alloimmunized mice received $100 \, mg \, kg^{-1}$ of cyclophosphamide i.p. Standard LAK and IL-2 treatment was given on days 3 to 7. The results after alloimmunization at day 5 are shown in Figure 4. The effect of IL-2 plus LAK cell treatment was abrogated in the mice immunized with the allogeneic splenocytes. In the allo-immunized mice that were pretreated with cyclophosphamide on day $-2$ the antitumour effect of LAK and IL-2 was retained. As is shown by the results in the appropriate controls, cyclophosphamide administered on day $-2$ had no effect on the growth of the syngeneic tumour. The i.p. administration of DBA/2J splenocytes at day $-10$ similarly diminished IL-2 plus LAK cell antitumour effects (see Table I: exp. 7A). Pretreatment with cyclophosphamide resulted in an identical restoration of the immunotherapeutic effects of IL-2 plus LAK (data not shown). These results suggest that the administration of cyclophosphamide eliminated the

**Figure 4** Pretreatment of alloimmunized mice with $100 \, mg \, kg^{-1}$ cyclophosphamide (CT) i.p. restores the anti-tumour effects of IL-2 plus LAK immunotherapy. BL/6 mice received $5 \times 10^8$ DBA/2J splenocytes i.p. 5 days prior to the i.p. inoculation of syngeneic MCA-105 tumour cells. IL-2 plus LAK cell immunotherapy was ineffective in alloimmunized mice. The administration of $100 \, mg \, kg^{-1}$ cyclophosphamide i.p. 2 days prior to the inoculation of MCA-105 restored the anti-tumour efficacy of IL-2 + LAK cell immunotherapy.

population of activated T-cells in the alloimmunized mice and thus eliminated the competition for IL-2 with the adoptively transferred LAK cells. Their activity and antitumour effect were therefore maintained.

### Discussion

These studies show that the antitumour effect of IL-2 in conjunction with the adoptive transfer of LAK cells in an i.p. tumour model is diminished in mice that are actively engaged in an immune response to an alloantigen. IL-2 is a lymphokine that can be used by different subpopulations of lymphocytes in order to expand and activate effector functions (Brooks *et al.*, 1985; Cheever *et al.*, 1984; Ettinghausen *et al.*, 1985*a*,*b*; Hefeneider *et al.*, 1983). The number of IL-2 receptors and the affinity of these receptors for IL-2 on a given lymphocyte population will determine the amount of IL-2 that can be bound and consumed. We hypothesize that the observed inactivation of IL-2 and LAK immunotherapy in alloimmunized mice is due to the consumption of IL-2 by the highly activated and expanded CTL population in these mice. Through a mechanism of competitive inhibition IL-2 will not be present in sufficient quantities in order to maintain LAK cell activity and as a result the antitumour effect is lost. The hypothesis that IL-2 consumption in recently alloimmunized mice is more rapid than in normal mice was corroborated by the finding that serum IL-2 levels following the administration of 20,000 units of IL-2 in these mice were lower than in nonimmunized mice after any given time interval. These *in vivo* experiments therefore corroborate the findings of our *in vitro* studies, which showed the impressive capacity of CTLs to absorb IL-2 and thereby inhibit the generation as well as the maintenance of LAK cell activity when these two cell populations were mixed in the presence of IL-2 (Sugarbaker *et al.*, 1986; and unpublished data). Since IL-2 rapidly upregulates the number of IL-2 receptors (Andrew *et al.*, 1984; Lipkowitz *et al.*, 1984) and has a strong proliferative effect on T-cells (Jacques *et al.*, 1986; Rubin *et al.*, 1985) we speculate that this rapidly expanding activated lymphocyte population absorbs the administered IL-2. Both the increased expression of IL-2 receptors on the cells as well as the soluble IL-2 receptors that are shed may be involved in the absorption of IL-2 (Hardt *et al.*, 1981; Rubin *et al.*, 1985) and thus play a part in the putative competitive inhibition model. The fact that LAK cell activity can be restored by administering high doses of IL-2 strongly supports this concept.

Other *in vitro* studies with alloactivated T-cells have focused on the suppressive effects these cells may have on cytotoxic T lymphocytes by absorption of IL-2 and/or through their rapid expansion in the presence of IL-2 (Fink *et al.*, 1984; Gunther *et al.*, 1982; Orosz & Ferguson, 1985; Palacios & Moller, 1981; Salomon *et al.*, 1984; Susskind *et al.*, 1983). D'Amore and Golub (1985) reported on the suppression of NK cell activity by a MLC generated population of NK-like cells and showed that this suppression was most likely caused by blocking of the target cells by this lymphocyte population. Alternatively, D'Amore and Golubs' data may suggest that the concentration of biological response modifiers in the microenvironment of the effector cell is extremely important to the continued function of that cell. Local-regional suppressor effects may be stronger than systemic suppressor effects.

The existence of soluble anti-IL-2 factors that are produced by CTL (Hardt *et al.*, 1981; Honda *et al.*, 1985) or the role of other suppressive factors (Kitamura *et al.*, 1984; Truit *et al.*, 1978) were found to play no role in our *in vitro* analysis (Sugarbaker, unpublished). In our tumour system the overriding cause for suppression seems to be competition for IL-2 by activated alloimmune cells. We thus postulate an alternative explanation as to the nature of suppression and suppressor cells.

It is of interest that suppression of IL-2 and LAK effects were shown to be eliminated by the administration of $100\,mg\,kg^{-1}$ cyclophosphamide 3 to 8 days after the allogeneic challenge. This dose of cyclophosphamide is known to abrogate an ongoing cytolytic T-cell response (Glasser, 1979) and has been shown to destroy T-cells cytolytic for P815 mastocytoma (North, 1985). The suppressor cell population is then presumably at the peak of its proliferative activity and most susceptible to cyclophosphamide (Turk & Poulter, 1972; Turk & Parker, 1982). The administration of cyclophosphamide two days prior to the inoculation of syngeneic tumour was shown to have no effect on tumour growth and the observed restoration of the antitumour effects of LAK and IL-2 therapy can be interpreted as a purely immunomodulative effect, presumably reflecting the elimination of the suppressor cell population as the source of IL-2 consumption. This effect of cyclophosphamide is reminiscent of the experimental work of North (1982). He showed that the adoptive immunotherapy with cytotoxic T lymphocytes was facilitated by the elimination of suppressor T-cells by cyclophosphamide.

Our competitive inhibition model for IL-2 offers an alternative interpretation for some of the immunosuppressive phenomena observed, but often not fully understood. It may lead to a reexamination and reinterpretation of some of the many immunosuppressive conditions that are known to exist and may lead us to the relevance of our observations with respect to the clinical situation in which a cancer patient is often involved in an active immune response to the sequelae of an operation, a blood transfusion or an infectious process. These conditions are known to be 'immunosuppressive' and this suppression may well in part be due to a consumption of biological response modifiers that otherwise could have been integrated into an antitumour directed immune response. In order to give immunotherapy an optimal chance, it may therefore be necessary to eliminate first these 'competitive conditions' mentioned above. The restoration of an immune response in mice with a chronic Trypanosoma Cruzi infection by IL-2 (Reed *et al.*, 1984), the impaired IL-2 production in granuloma bearing mice (Kobayashi *et al.*, 1985), the role of T-cells in abscess formatiom (Shapiro *et al.*, 1986), the beneficial effects of blood transfusions on the survival of kidney transplants (Opelz & Terasaki, 1980), the detrimental effect of blood transfusions on the five-year survival of patients with sarcoma (Rosenberg *et al.*, 1985*b*), breast, lung (Tartter *et al.*, 1983; 1984), or colorectal cancer (Agarwal & Blumberg, 1983; Burrows & Tartter, 1982), and the detrimental effect of a postoperative infection on the five-year survival rates after surgery for colorectal cancer (Nowacki & Szymendera, 1983) may all reflect the sequelae of an immunosuppressive state due to competition for IL-2 and/or other biological response modifiers. In order to obtain a maximal effect of the administration of IL-2 with a minimal dose of IL-2, which in view of the high dose IL-2 related toxicity (Lotze *et al.*, 1984; Matory *et al.*, 1985) is an important aspect, it may therefore be necessary to first eliminate competitive immune responses. Cyclophosphamide may be of value to attain this goal.

### References

AGARWAL, M. & BLUMBERG, N. (1983). Colon cancer patients transfused perioperatively have an increased incidence of recurrence. *Proc. Am. Soc. Blood Banks*, 419. (*Abstract*).

ANDREW, M.E., BRACIALE, V.L. & BRACIALE, T.J. (1984). Regulation of interleukin-2 receptor expression on murine cytotoxic T lymphocyte clones. *J. Immunol.*, **132**, 839.

ANDRIOLE, G.L., MULE, J.J., HANSEN, C.T., LINEHAN, W.M. & ROSENBERG, S.A. (1985). Evidence that activated killer cells and natural killer cells are distinct based on an analysis of congenitally immunodeficient mice. *J. Immunol.*, **135**, 2911.

BROOKS, C.G., HOLSCHER, M. & URDAL, D. (1985). Natural killer activity in cloned cytotoxic T lymphocytes regulation by interleukin-2, interferon, and specific antigen. *J. Immunol.*, **135**, 1145.

BURROWS, L. & TARTTER, P.I. (1982). Effect of blood transfusion on colonic malignancy recurrence rate. *Lancet*, **ii**, 662.

CHEEVER, M., GREENBERG, P.D., IRLE, C. & 5 others (1984). Interleukin-2 administered *in vivo* induces the growth of cultured T-cells *in vivo*. *J. Immunol.*, **132**, 2259.

D'AMORE, P.J. & GOLUB, S.H. (1985). Suppression of human NK cell cytotoxicity by an MLC-generated cell population. *J. Immunol.*, **134**, 272.

ETTINGHAUSEN, S.E., LIPFORD, E.H. & MULE, J.J. (1985*a*). Systemic administration of recombinant interleukin-2 stimulates *in vivo* lymphoid cell proliferation in tissues. *J. Immunol.*, **135**, 1488.

ETTINGHAUSEN, S.E., LIPFORD, E.H., MULE, J.J. & ROSENBERG, S.A. (1985*b*). Recombinant interleukin-2 stimulates the *in vivo* proliferation of adoptively transferred lymphokine-activated killer (LAK) cells. *J. Immunol.*, **135**, 3623.

FINK, P.J., RAMMENSEE, H.G., BENEDETTO, J.D., STAERZ, U.D., LEFRANCOIS, L. & BEVAN, M.J. (1984). Studies on the mechanism of suppression of primary cytotoxic responses by cloned cytotoxic T lymphocytes. *J. Immunol.*, **133**, 1769.

GLASSER, M. (1979). Regulation of specific cell-mediated cytotoxic response against SV40000-induced tumor associated antigens by depletion of suppressor T-cells with cyclophosphamide in mice. *J. Exp. Med.*, **149**, 774.

GUNTHER, J., HAAS, W. & VONBOEHMER, H. (1982). Suppression of T-cell responses through competition for T-cell growth factor (interleukin-2). *Eur. J. Immunol.*, **12**, 247.

HARDT, C., ROLLINGHOFF, M., PFIZENMAIER, K., MOSMANN, H. & WAGNER, H. (1981). Lyt-23 cyclophosphamide sensitive T-cells regulate the activity of an interleukin-2 inhibitor *in vivo*. *J. Exp. Med.*, **154,** 262.

HEFENEIDER, S.T., CONLON, P.J., HENNEY, C.S. & GILLIS, S. (1983). *In vivo* interleukin-2 administration augments the generation of alloreactive cytolytic T lymphocytes and resident natural killer cells. *J. Immunol.*, **130,** 222.

HOLLANDER, M. & WOLFE, D. (1973). (eds) In *Nonparametric statistical methods*. p. 114. New York, John Wiley and Sons.

HONDA, M., CHAN, C. & SHEVACH, E. (1985). Characterization and partial purification of a specific interleukin-2 inhibitor. *J. Immunol.*, **135,** 1834.

JACQUES, Y., LE MAUFF, B., GODARD, A., OLIVE, D., MOREAU, J.F. & SOULILLOU, J.P. (1986). Regulation of interleukin-2 receptor expression on a human T lymphocyte clone, synergism between alloantigeneic stimulation and interleukin-2. *J. Immunol.*, **136,** 1693.

KITAMURA, K., NAKAUCHI, H., KOYASU, S., YAHARA, I., OKUMURA, K. & TADA, T. (1984). Characterization of an antigen-specific suppressive factor derived from a cloned suppressor effector T-cell line. *J. Immunol.*, **133,** 1371.

KOBAYASHI, K., ALLRED, C. & YOSHIDA, T. (1985). Mechanisms of suppressed cell mediated immunity and impaired antigen-induced interleukin-2 production in granuloma-bearing mice. *J. Immunol.*, **135,** 2996.

LAFRENIERE, R. & ROSENBERG, S.A. (1985). Successful immunotherapy of murine experimental hepatic metastases with lymphokine-activated killer cells and recombinant interleukin-2. *Cancer Res.*, **45,** 3735.

LIPKOWITZ, S., GREENE, W.C., RUBIN, A.L., NOVOGRODSKY, A. & STENZEL, K.H. (1984). Expression of receptors for interleukin-2: Role in the commitment of T lymphocytes to proliferate. *J. Immunol.*, **132,** 31.

LOTZE, M.T., ROBB, R.J., SHARROW, S.O., FRANA, L.W. & ROSENBERT, S.A. (1984). Systemic administration of interleukin-2 in humans. *J. Biol. Res. Mod.*, **3,** 475.

MATORY, Y.L., CHANG, A.E., LIPFORD, E.H., BRAZIEL, R., HYATT, C.L. & ROSENBERT, S.A. (1985). The toxicity of recombinant human interleukin-2 in rats following intravenous infusion. *J. Biol. Res. Mod.*, **4,** 377.

MULE, J.J., SHU, S., SCHWARZ, S.L. & ROSENBERG, S.A. (1984). Adoptive immunotherapy of established pulmonary metastases with LAK cells and recombinant interleukin-2. *Science*, **255,** 1487.

MULE, J.J., SHU, S. & ROSENBERG, S.A. (1985). The anti-tumor efficacy of lymphokine activated killer cells and recombinant interleukin-2 *in vivo*. *J. Immunol.*, **135,** 646.

NORTH, R.J. (1982). Cyclophosphamide-facilitated adoptive immunotherapy of an established tumor depends on elimination of tumor-induced suppressor T cells. *J. Exp. Med.*, **155,** 1063.

NORTH, R.J. (1985). The murine antitumor immune response and its therapeutic manipulation. *Adv. Immunol.*, **35,** 89.

NOWACKI, N.P. & SZYMENDERA, J.J. (1983). The strongest prognostic factors in colorectal carcinoma: Surgicopathological stage of disease and postoperative fever. *Dis. Colon Rectum*, **26,** 263.

OPELZ, G. & TERASAKI, P.I. (1980). Dominant effect of transfusions on kidney graft survival. *Transplantation*, **29,** 153.

OROSZ, C.G. & FERGUSON, R.M. (1985). Suppression of *in vitro* CML generation by alloactivated lymphocytes: Analysis of antigen-nonspecific suppressive mechanisms. *J. Immunol.*, **134,** 45.

PALACIOS, R. & MOLLER, G. (1981). T-cell growth factor abrogates concanavalin A-induced suppressor cell function. *J. Exp. Med.*, **153,** 1360.

REED, S.G., INBERSO, J.A. & ROTERS, S.B. (1984). Suppressed antibody responses to sheep erythrocytes in mice with chronic Trypanosoma Cruzi infections are restored with interleukin-2. *J. Immunol.*, **133,** 3333.

ROSENBERG, S.A., GRIMM, E.A., McGROGAN, M. & 4 others (1984). Biological activity of recombinant human interleukin-2 produced in *E. coli*. *Science*, **223,** 1412.

ROSENBERG, S.A., LOTZE, M.T., MUUL, L.M. & 10 others (1985*a*). Observations on the systemic administration of autologous activated killer cells and recombinant interleukin-2 to patients with metastatic cancer. *New Engl. J. Med.*, **313,** 1485.

ROSENBERG, S.A., SEIPP, S.A., WHITE, D.E. & WESLEY, R. (1985*b*). Perioperative blood transfusions are associated with increased rates of recurrence and decreased rates of survival in patients with high-grade soft tissue sarcomas of the extremities. *J. Clin. Oncol.*, **3,** 698.

ROSENBERG, S.A. (1986). The adoptive immunotherapy of cancer using the transfer of activated lymphoid cells and interleukin-2. *Sem. Oncol.*, **13,** 200.

RUBIN, L.A., KURMAN, C.C., FRITZ, M.E. & 4 others (1985). Soluble interleukin-2 receptors are released from activated human lymphoid cells *in vitro*. *J. Immunol.*, **135,** 3172.

SALOMON, D.R., COHEN, D.J., WILLIAMS, J.M. & CARPENTER, C.B. (1984). T-cell synergy in the primary MLR: Proliferative kinetics, effector cell generation, and IL-2 production. *J. Immunol.*, **133,** 3075.

SHAPIRO, M.E., KASPER, D.L., ZALEZNIK, D.F., SPRIGGS, S., ONDERKOND, A.B. & FINBERG, R.W. (1986). Cellular control of abscess formation: Role of T-cells in the regulation of abscesses formed in response to *Bacteroides Fragilis*. *J. Immunol.*, **137,** 341.

SUGARBAKER, P.H., EGGERMONT, A.M.M., STELLER, E.P. & MATTHEWS, W. (1986). Cytotoxic T lymphocytes (CTL) compete with lymphokine activated killer (LAK) cells for interleukin-2 (IL-2) and can abrogate their effects. *Proc. Am. Assoc. Cancer Res.*, **27,** 343. (*Abstract*).

STELLER, E.P., OTTOW, R.T., MATTHEWS, M., SUGARBAKER, P.H. & ROSENBERG, S.A. (1985). Recombinant Interleukin-2 and adoptively transferred lymphokine-activated killer cells in the treatment of experimental peritoneal carcinomatosis. *Surg. Forum.*, **36,** 390.

SUSSKIND, B.M., MERLUZZI, V.J., FAANES, R.B., PALLADINA, M.A. & SUNG CHOI, Y. (1983). Regulatory mechanisms in cytotoxic T lymphocyte development. I. A suppressor T-cell subset that regulates the proliferative stage of CTL development. *J. Immunol.*, **130,** 527.

TARTTER, P.I., PAPATESTAS, A.E. & LESNICK, G. (1983). Breast cancer recurrence is associated with perioperative blood transfusions. *Proc. Am. Soc. Clin. Oncol.*, **2,** 50. (*Abstract*).

TARTTER, P.I., BURROWS, L. & KIRSCHNER, P.A. (1984). Perioperative blood transfusion adversely affects prognosis after resection of stage I (NO) non-oat cell lung cancer. *Am. Assoc. Thorac. Surg. Proc.*, **88,** 659.

TRUIT, G.A., RICH, R.R. & SOLLIDAY, R.S. (1978). Suppression of cytotoxic lymphocyte responses *in vitro* by soluble products of alloantigen-activated spleen cells. *J. Immunol.*, **121,** 1045.

TURK, J.L. & POULTER, L.W. (1972). Selective depletion of lymphoid tissue by cyclophosphamide. *Clin. Exp. Immunol.*, **10,** 285.

TURK, J.L. & PARKER, D. (1982). Effect of cyclophosphamide on immunological control mechanisms. *Immunol. Rev.*, **65,** 99.